# MetaPathways: a modular pipeline for constructing pathway/genome databases from environmental sequence information

**DOI:** 10.1186/1471-2105-14-202

**Published:** 2013-06-21

**Authors:** Kishori M Konwar, Niels W Hanson, Antoine P Pagé, Steven J Hallam

**Affiliations:** 1 Department of Microbiology & Immunology, University of British Columbia, Vancouver, BC V6T1Z3, Canada; 2 Graduate Program in Bioinformatics, University of British Columbia, Vancouver, BC Canada

**Keywords:** Environmental pathway/Genome Database (ePGDB), Metagenome, Pathway tools, PathoLogic, MetaCyc, Microbial community, Metabolism, Metabolic interaction networks

## Abstract

**Background:**

A central challenge to understanding the ecological and biogeochemical roles of microorganisms in natural and human engineered ecosystems is the reconstruction of metabolic interaction networks from environmental sequence information. The dominant paradigm in metabolic reconstruction is to assign functional annotations using BLAST. Functional annotations are then projected onto symbolic representations of metabolism in the form of KEGG pathways or SEED subsystems.

**Results:**

Here we present MetaPathways, an open source pipeline for pathway inference that uses the PathoLogic algorithm to map functional annotations onto the MetaCyc collection of reactions and pathways, and construct environmental Pathway/Genome Databases (ePGDBs) compatible with the editing and navigation features of Pathway Tools. The pipeline accepts assembled or unassembled nucleotide sequences, performs quality assessment and control, predicts and annotates noncoding genes and open reading frames, and produces inputs to PathoLogic. In addition to constructing ePGDBs, MetaPathways uses MLTreeMap to build phylogenetic trees for selected taxonomic anchor and functional gene markers, converts General Feature Format (GFF) files into concatenated GenBank files for ePGDB construction based on third-party annotations, and generates useful file formats including Sequin files for direct GenBank submission and gene feature tables summarizing annotations, MLTreeMap trees, and ePGDB pathway coverage summaries for statistical comparisons.

**Conclusions:**

MetaPathways provides users with a modular annotation and analysis pipeline for predicting metabolic interaction networks from environmental sequence information using an alternative to KEGG pathways and SEED subsystems mapping. It is extensible to genomic and transcriptomic datasets from a wide range of sequencing platforms, and generates useful data products for microbial community structure and function analysis. The MetaPathways software package, installation instructions, and example data can be obtained from http://hallam.microbiology.ubc.ca/MetaPathways.

## Background

Metabolic interactions between microorganisms direct matter and energy transformations integral to ecosystem function [1-3]. Plurality sequencing methods enable exploration of potential (metagenomic) and expressed (metatranscriptomic) metabolic interactions with the aid of computational methods that assemble or cluster contiguous reads, search for patterns or motifs representing genes, and reconstruct pathways from environmental sequence information [4-6]. The prevailing paradigm in pathway reconstruction is to assign functional annotation based on sequence homology using BLAST [7]. Functional annotations are then projected onto symbolic representations of metabolism such as KEGG pathways [8-10] or SEED subsystems [11] revealing network structure.

With the expansion of next generation sequencing technologies, increasingly complex datasets are being generated for thousands of environmental samples resulting in analytic bottlenecks with the potential to stymie pathway reconstruction efforts. As a result, on-line services for metabolic reconstruction have been developed to externalize data processing burdens and provide warehousing and visualization tools for environmental sequence information. Popular on-line services for metabolic reconstruction include Integrated Microbial Genomes and Metagenomes (IMG/M), Community Cyberinfrastructure for Advanced Microbial Ecology Research and Analysis (CAMERA), and Metagenome Rapid Annotation using Subsystem Technology (MG-RAST). Both IMG/M [12,13] and CAMERA [14] warehouse public datasets and provide management, exploration, and visualization tools for environmental sequence information. MG-RAST [15,16] warehouses public datasets and provides gene prediction and annotation services based on SEED subsystems mapping using FIGfams [17] and BLAST. While on-line services increase access to computational resources, idiosyncratic data processing and management practices common to each service insulate users from command-line optimization and create formatting and data transfer restrictions.

Pathway Tools [18,19] is a production-quality software system that enables construction, management and navigation of symbolic representations of metabolism in the form of Pathway/Genome databases (PGDBs). A PGDB encodes contemporary knowledge about the network properties of a cellular organism. Pathway Tools supports four modular operations including metabolic pathway prediction using PathoLogic [18,20], metabolic flux modeling using MetaFlux [21], PGDB editing and navigation tools including manual or automated search functions, and comparative analysis and systems level visualizations. Further, genes, reactions, and pathways can be exported via the Systems Biology Markup Language (SMBL) framework, allowing interoperability and downstream analysis with compatible systems biology tools [22]. The Pathologic module allows users to construct new PGDBs from an annotated genome using MetaCyc [23,24], a highly curated, non-redundant and experimentally validated database of metabolic pathways representing all domains of life. Unlike KEGG pathways or SEED subsystems, MetaCyc emphasizes smaller, evolutionary conserved units of metabolism or pathway variants that are regulated and transferred together. MetaCyc is also extensively commented with pathway descriptions, literature citations, and enzyme properties including subunit composition, substrate specificity, cofactors, activators, and inhibitors each connected to specific pathway variants. A web-server version of the Pathway Tools editing and navigation tools supports on-line browsing, manual curating and web publishing of PGDBs. Currently PGDBs for 2037 cellular organisms have been constructed and incorporated into the BioCyc collection [25].

Here we extend the PGDB concept for cellular organisms to microbial community structure and function through the introduction of MetaPathways, a modular pipeline for pathway inference that uses the PathoLogic algorithm to build environmental PGDBs (ePGDBs) compatible with the editing and navigation features of Pathway Tools. The pipeline accepts assembled contig or unassembled nucleotide sequences, performs quality control and coverage estimates, predicts and annotates noncoding genes and open reading frames, and produces concatenated GenBank files used as inputs to PathoLogic. In addition to constructing ePGDBs, MetaPathways uses MLTreeMap [26] to build phylogenetic trees for selected taxonomic anchor and functional gene markers, converts General Feature Format (.gff) files into concatenated GenBank (.gbk) files for ePGDB construction using third-party annotations, and generates useful file formats including Sequin files for direct GenBank submission and gene feature tables summarizing annotations, MLTreeMap, and ePGDBs for statistical comparisons.

## Implementation

**Figure 1:**
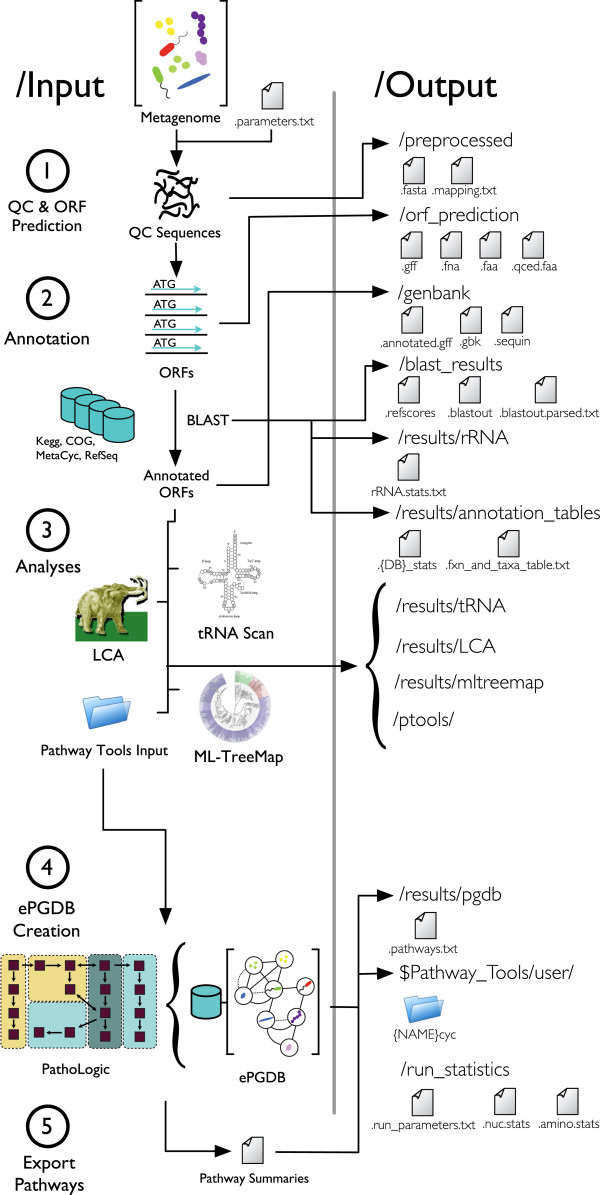
**The MetaPathways pipeline consists of five operational stages including (1) Quality control (QC) and open reading frame (ORF) prediction (2) ORF annotation, (3) Modular analysis (4) ePGDB construction, and (5) Pathway Export.** Inputs and executables are depicted on the left with corresponding output directories and exported files on the right.

**Figure 2:**
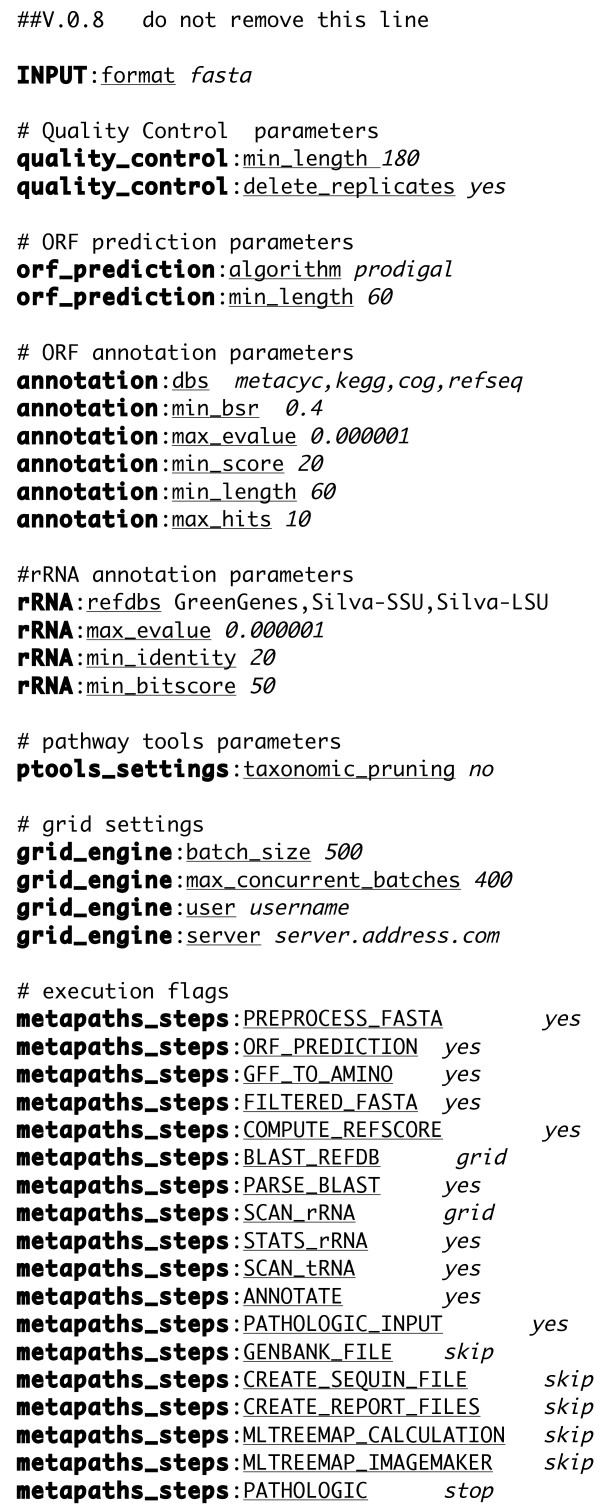
**The parameter file (.parameters.txt) delimits software settings for successive operational stages and can be easily edited to enable or disable specific operations or modify default settings associated with specific software components.** Execution flags including yes, skip, and grid control successive pipeline operations.

MetaPathways is a modular pipeline written in Python that calls software components written in C/C++, Perl, and Python. Required input files for MetaPathways include metagenomic or metatranscriptomic sequence data in one of several file formats (.fasta, .gff, or .gbk). The pipeline consists of five operational stages including (1) Quality control (QC) and open reading frame (ORF) prediction (2) ORF annotation, (3) Modular analysis (4) ePGDB construction, and (5) Pathway Export (Figure 1). A parameter file (.parameters.txt) delimits software settings for successive operational stages and can be easily edited to enable or disable specific operations or modify default settings associated with specific software components (Figure 2).

### Quality control & ORF prediction

Sequence information is processed to remove sequences below a user-defined length threshold, incompletely specified bases are converted to the character ‘N’ and input sequence identifiers are sequentially renamed (i.e., sample_#). A mapping file (.mapping.txt) is exported relating original input sequence names with sequential names. ORFs are predicted using the Prokaryotic Dynamic Programming Genefinding Algorithm (Prodigal), which can detect incomplete or fragmentary ORFs [27]. Coordinate information, nucleotide, and conceptually translated amino acid sequences for predicted ORFS are exported as .gff, .fna, and .faa files, respectively. By default, ORFs below a default length of 180 nucleotides or 60 amino acids are removed (.qced.faa) and nucleotide (.nuc.stats) and amino acid sequence (.amino.stats) distribution summaries before and after post-processing are exported.

### ORF annotation

Conceptually translated ORFs are queried against user-defined reference protein databases including KEGG [8], COG [28], RefSeq [29], and MetaCyc, where MetaCyc refers to the pathway hole-filler database included with Pathway Tools [19], using the protein BLAST or optimized LAST algorithm [30] in tabular format (.blastout/.lastout). Concomitant with reference protein database queries, self-BLAST bit-scores are calculated (.refscores) enabling a measure of similarity using the BLAST-score ratio (BSR) [31]. BLAST summary tables parsing resulting e-values, percent identities, bit-scores, lengths, and BSRs are exported for each reference database (.blastout.parsed.txt) highlighting the e-value, percent identity, bit-score, length, and BSR values. By default, annotations with BSRs below 0.4 corresponding to the so-called “Twilight Zone” of gene annotation [32] are excluded from summary tables.

BLAST represents a computational burden that can limit pipeline performance on big datasets when implemented on local machines. Therefore, we have adopted a representational state transfer (REST) design supporting implementation on external Sun Grid Engine servers or supercomputers [33]. A user-defined connection filter (username, password and external server address for configuration) and externalization script enables setup (uploading, formatting, and installing BLAST databases and executables), parallel splitting of BLAST jobs, queue submission and management, and the collection and consolidation of results back to the local machine. This creates a RESTful system that is robust to unforeseen interruption and is readily transferable to the cloud. MetaPathways can also incorporate third party annotations sourced from .gbk or .gff files directly using embedded file-interconversion scripts.

Tabular BLAST results returned from local or external resources are used to assign product descriptions to predicted ORFs based on an internal heuristic to standardize product descriptions. For each ORF, the top e-value from each reference database is selected and given an “information score” based on the number of distinct enzymatic words and a preference to Enzyme Commission (EC) numbers (+10 score). Functional annotations with the highest information score are appended to the ORF description and exported as a tabular file (.annotated.gff). Predicted ORFs with no BLAST hits are annotated as “hypothetical protein.” In addition, BLAST summaries of functional annotations at different hierarchical levels (Cite KEGG/COG) are exported for KEGG and COG databases (.{DB}.stats.txt). Following functional annotation of predicted ORFs, nucleotide sequences are queried against reference nucleotide databases including SILVA [34] and GreenGenes [35] to identify ribosomal RNA genes. BLAST summary tables containing e-values, percent identities, bit-scores, lengths and taxonomic identity are exported for each reference database (.rRNA.stats.txt). This information is combined with the file .annotated.gff to generate input files for ePGDB construction, standard .gbk file and .sequin file for NCBI submission.

### Analyses

MetaPathways currently implements three modular analyses using existing or derived files as input (.fasta, .gbk, or .gff input formats and derived tabular results). The first analytic module implements tRNA-scan (version 1.4) to identify relevant tRNAs from QC nucleotide sequences [36]. Resulting tRNA identifications are appended to the .gbk and .sequin files as additional annotations. The second analytic module implements the popular and widely accepted LCA algorithm for taxonomic binning [37]. The lowest common ancestor in the NCBI taxonomic hierarchy is selected based on the previously calculated BLAST-hits from the RefSeq database. This effectively sums the number of BLAST hits at the lowest shared position of the hierarchy. The RefSeq taxonomic names often contain multiple synonyms or alternative spellings. Therefore, names that conform to the official NCBI taxonomy are selected in preference over unknown synonyms. The third analytic module implements MLTreeMap (version 2.061) to identify and construct trees for selected phylogenetic and functional marker genes from QC nucleotide sequences [26]. Results from LCA and MLTreeMap analysis are exported as a tabular file (fxn_and_taxa_table.txt). Additional analysis modules implemented from the command line can be directly inserted into the pipeline. By convention, results from each analysis are placed in a self-titled directory under the parent results directory (i.e. /results/mltreemap).

### ePGDB construction

The annotated ORF file (.annotated.gff) is parsed and separated into four files including (1) an annotation file containing gene product information, (2) a nucleotide sequence file in .fasta format, (3) a genetic-elements file, and (4) a PGDB parameters file (/ptools/). For the purposes of ePGDB construction, nucleotide input files are concatenated to form a single “chromosomal” element defining a composite genome. Concatenation is necessary to improve Pathway Tools performance on input files containing thousands of genetic-elements in batch mode. PathoLogic uses these input files to predict metabolic pathways based on defined biochemical rules (pathway completion, diagnostic/key enzymes, biosynthesis and degradation constraints) resulting in ePGDB construction and export to the local Pathway Tools internal library ($Pathway_Tools/user/).

**Figure 3:**
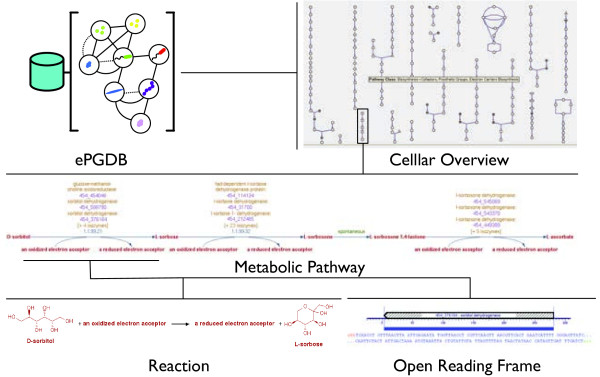
**Environmental PGDBs (ePGDBS) and their contents are accessible through a built-in web server, allowing the knowledge of genes, proteins, metabolic and regulatory networks embedded within them to be queried, compared, curated and shared in a distributed fashion via the Internet.** The “Cellular Overview” feature displays ePGDB contents in the form of interactive glyphs that link sub-pathways together in a global picture of metabolism scalable down to the level of pathways, reactions and individual open reading frames.

**Figure 4:**
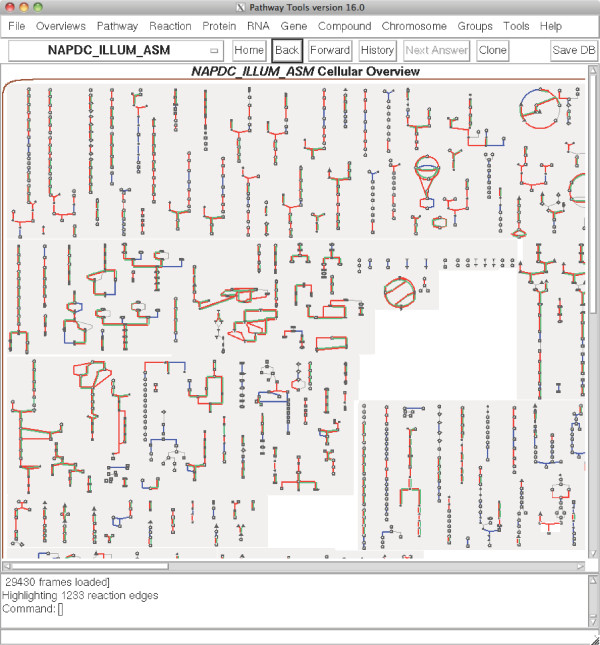
**Cellular overview in the Pathway Tools software highlighting pathways found in the Naphtha-degrading culture sample.** Using the assembled Illumina pathways as a backbone (blue), common predicted pathways from the 454 (red) and Sanger (green) sequencings are placed on top. Allowing exploration of pathways predicted using different sequence technologies and depth.

Environmental PGDBs and their contents are accessible, internally or externally, through a built-in web server, allowing the knowledge of genes, proteins, metabolic and regulatory networks embedded within them to be queried, compared, curated and shared in a distributed fashion via the Internet. In addition to powerful search and retrieval functions, Pathway Tools provides a metabolic encyclopedia, based on primary literature citations encompassing more than 1900 evolutionary conserved sub-pathways within the MetaCyc schema [21,24,38]. The “Cellular Overview” feature displays ePGDB contents in the form of interactive glyphs that link sub-pathways together in a global picture of metabolism [39]. Hovering over a glyph activates a tooltip that identifies the pathway and clicking on a glyph reveals pathway interactions at the level of enzymes, reactions and identified ORFs (Figure 3). Direct comparisons between ePGDBs can be made using coloured overlays on the cellular overview revealing similarities and differences in metabolic pathway composition (Figure 4).

### Pathway export

Information is extracted from ePGDBs including ORF identities, enzyme abundance, and pathway coverage and exported in tabular format (.pathways.txt, and pathway_rxns.txt). A receipt and time-stamp for each successful pipeline execution is created containing the specific parameter settings used in ePGDB creation (.run_parameters.txt).

### Performance

MetaPathways performance was evaluated using unassembled (Sanger fosmid end, 454 pyrosequencing) and assembled (Illumina HiSeq) genomic sequence information sourced from a naphtha-degrading, methanogenic enrichment culture (Additional file 1). Input datasets captured a range of nucleotide sequence numbers, lengths and sample coverage. Base pathway prediction and runtime increased as a function of nucleotide sequence number. While runtime complexity varies in relation to input file size and external resource allocation, empirical runtimes approached an upward limit of 2,300 sequences per minute, when externalizing BLAST on the Western Canadian Research Grid [40] (Additional file 1). Remaining analyses and data transformations were performed locally on a Mac Pro desktop computer running Mac OSX 10.6.8 with a 2×2.4 Ghz Quad-Core Intel Xeon processors and 24GB of 1066Mhz DDR3 RAM.

#### Evaluation of pathway prediction with simulated metagenomes

**Figure 5:**
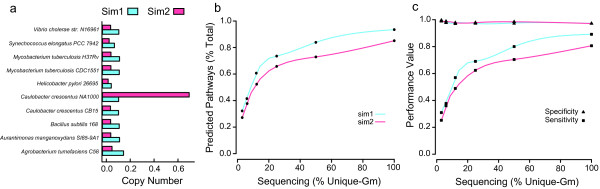
**Analysis on in silico simulated sequencing experiments across different levels of coverage and taxon distribution.** Sim1 (blue) contains ten tier-2 PGDB genomes in approximately equal proportion. Sim2 (red) has one taxon overrepresented by 20-fold. Tier-2 taxa were selected on the basis of approximately equal genome size and gene content (**a**). Predicted pathway recovery as a percentage of the total pathways predicted from the full genome (**b**). Specificity (triangles) and sensitivity (squares) classification performance of predicted pathways using the pathways predicted on the full genomes as the gold standard (**c**). Interpolating lines were drawn via a natural spline.

Previous studies have evaluated PathoLogic’s performance on fully-sequenced genomes establishing its pathway prediction power in relation to machine learning methods [41]. To determine PathoLogic’s performance on combined and incomplete genomes sourced from environmental sequence information we generated simulated metagenomes from 10 BioCyc tier-2 PGDBs (Additional file 2) using MetaSim [42] (Sanger sequencing, average length 700 bp, standard deviation 100 bp) with differing sequence coverage and taxon distribution profiles (Sim1 and Sim2). Tier-2 PGDBs were selected to minimize potential name mapping errors between MetaPathways’ annotations and extant MetaCyc annotations [41]. In Sim1 each genome was present at equal coverage and in Sim2 the *Caulobacter crescentus* NA1000 genome was overrepresented by 20-fold (Figure 5a). Simulations manifesting progressively larger fractions of total unique sequence length (unique-Gm) revealed that pathway recovery increases with sequence coverage (Figure 5b). Specificity, a measure of the confidence in accurate pathway prediction was high (>85%) regardless of taxonomic distribution or sequence coverage (Figure 5c) consistent with reduced Type I errors (false positives). However, sensitivity, a measure of the confidence in predicting specific pathways present in the sample, was reduced at low coverage consistent with increased Type II errors (false negatives) (Figure 5c). A 6% reduction in pathway recovery between Sim1 and Sim2 was observed, suggesting that pathway prediction follows a collector’s curve in which core metabolic functions shared between community members initially accumulate. As coverage increases, the encounter frequency for accessory genes increases resulting in improved pathway prediction approaching a limit based on extant MetaCyc pathways. Summary statistics including F-measure and Matthews Correlation Coefficient that balance between Type I and Type II errors, reinforce the observation that PathoLogic’s performance improves with increasing sequence coverage (Table 1 and Additional file 3).

**Table 1:** Pathway classification performance statistics for simulated metagenomes Sim1 and Sim2 at progressively larger sequence coverage

**Sample**	**Gm**	**Precision**	**Sensitivity**	**Specificity**	**Accuracy**	**F-measure**	**Matthews**
Sim1	(1/32)	0.96	0.31	0.99	0.73	0.47	0.79
Sim1	(1/16)	0.70	0.38	0.98	0.75	0.53	0.73
Sim1	(1/8)	0.76	0.57	0.98	0.82	0.71	0.81
Sim1	(1/4)	0.85	0.69	0.97	0.86	0.80	0.83
Sim1	(1/2)	0.81	0.80	0.98	0.91	0.87	0.88
Sim1	(1/1)	0.84	0.89	0.97	0.94	0.92	0.91
Sim2	(1/32)	0.93	0.25	0.99	0.70	0.40	0.74
Sim2	(1/16)	0.95	0.36	0.99	0.75	0.53	0.78
Sim2	(1/8)	0.93	0.49	0.98	0.79	0.64	0.78
Sim2	(1/4)	0.95	0.62	0.98	0.84	0.75	0.83
Sim2	(1/2)	0.97	0.70	0.98	0.88	0.81	0.87
Sim2	(1/1)	0.95	0.81	0.97	0.91	0.87	0.87

### Related work

While efforts to model microbial community structure in relation to environmental parameters have successfully predicted real-world distribution and diversity patterns in the surface ocean [43-45], the extension of modeling approaches to microbial metabolic interaction networks remains nascent. Function-based models such as Predicted Relative Metabolic Turnover (PRMT) predict metabolic flux in the environment based on the abundance of unique functional annotations using MG-RAST [46]. More recently, Abubucker and colleagues developed the Human Microbiome Project Unified Metabolic Analysis Network (HUMAnN) for metabolic reconstruction [47]. HUMAnN integrates MinPath to reconcile the multiple mapping problem associated with BLAST-based annotations for metabolic inference based on KEGG pathways and SEED subsystems [48] with additional taxonomic limitation and gap filling algorithms to reduce false positives and correct for rare genes in abundant pathways. HUMAnN results have been compared using Metagenomics Reports (METAREP) data storage and retrieval pipeline that supports scalable and dynamic analysis of complex environmental datasets [49]. While Pathway Tools uses its set of biochemical rules for pathway prediction, an alternative to Pathway Tools for the construction of genome-scale metabolic networks has also been integrated into SEED servers. This approach projects reactions onto the comparatively coarser KEGG metabolic map without further filtering or weighting results, and applies a mixed linear integer optimization for filling reaction gaps [50,51]. However, this method has not yet been applied to metabolic interaction networks in the environment.

### Pipeline limitations

Compared to current methods that project functional annotations from environmental sequence information onto KEGG pathways or SEED subsystems, MetaPathways enables an alternative algorithmic approach to metabolic reconstruction using evolutionarily conserved pathway prediction based on coverage and biochemical pathway rules. Moreover, the pipeline performs taxonomic binning and functional gene annotation, integrates external resource partitioning on compute clusters using the Sun Grid engine, and supports useful data transformation and formatting options. While we have demonstrated pipeline scalability with next generation sequencing datasets, further improvements to computationally intensive stages including BLAST/LAST-based annotation and ePGDB construction are needed to keep pace with projected advances in sequencing throughput. Future pipeline implementations will enable users to harness multi-core desktop computers to build local grid engines or to externalize BLAST and ePGDB construction on commercial computing resources such as the Amazon Elastic Compute Cloud (EC2). As an alternative to comprehensive all-against-all homology searches, future pipeline implementations will also incorporate scalable and distributed clustering algorithms enabling functional annotation based on hierarchical cluster assignments [17,52-54].

Aside from runtime improvements, additional data transformation and visual analysis modules expanding on existing taxonomic binning and marker gene identification components are needed. These include coverage statistics for assembled sequence information, data matrices and interactive visualizations indicating numerical abundance and taxonomic distribution of enzymatic steps, self-organizing maps and automated methods to append single cell or population genome assemblies to the NCBI hierarchy for more accurate taxonomic binning. Additional reference databases for 5S, 7S and 23S RNA genes and updates to the current MetaCyc database that include more biogeochemically relevant pathways are needed to improve BLAST and cluster-based annotation efforts. Finally, more experience and operational insight is needed in constructing, comparing and interpreting ePGDBs to identify potential sources of error and inform ongoing Pathway Tools development efforts.

## Conclusions

MetaPathways provides users with a modular annotation and analysis pipeline for predicting metabolic interaction networks from environmental sequence information. It is extensible to genomic and transcriptomic datasets from multiple sequencing platforms, and generates useful data products for microbial community structure and functional analysis including phylogenetic trees, taxonomic bins and tabular annotation files. The pipeline provides local and external computing solutions for implementing BLAST/LAST homology searches, resolves data handling issues associated with .gbk and .gff file conversion and NCBI submission, and generates ePGBDs using Pathway Tools for pathway inference and interactive visualization. The MetaPathways software, installation instructions, tutorials and example data can be obtained from http://github.com/hallamlab/MetaPathways/ or http://hallam.microbiology.ubc.ca/MetaPathways.

## Availability and requirements

**Project Name:** MetaPathways 1.0.

**Project Home Page:**http://hallam.microbiology.ubc.ca/MetaPathways.

**Operating system(s):** Linux/Unix, Mac OSX 10.6.x or later, Windows XP.

**Programming Languages:** C/C++, Python 2.7+, Perl.

**Other Requirements:** GCC compiler, NCBI BLAST 2.2.25+, Pathway Tools 16.0.

**License:** GNU GPL, Academic licenses needed for BLAST and Pathway Tools.

## Abbreviations

ePGDB: Environmental Pathway/Genome Database; ORF: Open reading frame; EC: Enzyme commission; REST: Representational state transfer.

## Competing interests

The authors are unaware of any competing interests.

## Authors’ contributions

KMK was the primary pipeline developer and co-wrote the paper. NWH assisted with pipeline development and co-wrote the paper. APP helped conceptualize and implement initial pipeline implementations. SJH conceived pipeline architecture, provided essential feedback on data products, integration and formatting, and co-wrote the paper. All authors read and approved the final manuscript.

## Supplementary Material

Additional file 1 MetaPathways validation summary based on a comparison of three sequencing methods on a common sample. Click here for file

Additional file 2 Source genome statistics for simulated metagenomes sim1 and sim2. Click here for file

Additional file 3 Confusion tables for classification analysis of simulated metagenomes sim1 and sim2 at progressively larger sequence coverage. Click here for file
